# TRPM channels are required for rhythmicity in the ultradian defecation rhythm of *C. elegans*

**DOI:** 10.1186/1472-6793-8-11

**Published:** 2008-05-21

**Authors:** Claire SM Kwan, Rafael P Vázquez-Manrique, Sung Ly, Kshamata Goyal, Howard A Baylis

**Affiliations:** 1 Department of Zoology, University of Cambridge, Downing Street, Cambridge, CB2 3EJ, UK

## Abstract

**Background:**

Ultradian rhythms, rhythms with a period of less than 24 hours, are a widespread and fundamental aspect of life. The mechanisms underlying the control of such rhythms remain only partially understood. Defecation in *C. elegans *is a very tightly controlled rhythmic process. Underlying the defecation motor programme is an oscillator which functions in the intestinal cells of the animal. This mechanism includes periodic calcium release and subsequent intercellular calcium waves which in turn regulate the muscle contractions that make up the defecation motor programme. Here we investigate the role of TRPM cation channels in this process.

**Results:**

We use RNA interference (RNAi) to perturb TRPM channel gene expression. We show that combined knock down of two of the TRPM encoding genes, *gon-2 *and *gtl-1*, results in an increase in the variability of the cycle but no change in the mean, in normal culture conditions. By altering the mean using environmental (temperature) and genetic approaches we show that this increase in variability is separable from changes in the mean. We show that *gon-2 *and *gtl-1 *interact with components of the calcium signalling machinery (*itr-1 *the *C. elegans *inositol 1,4,5-trisphosphate receptor) and with plasma membrane ion channels (*flr-1 *and *kqt-3*) which are known to regulate the defecation oscillator. Interactions with these genes result in changes to the mean period and variability. We also show that knocking down a putative transcription factor can suppress the increased variability caused by reduction of *gon-2 *and *gtl-1 *function. We also identify a previously unrecognised tendency of the defecation cycle to compensate for cycles with aberrant length by adjusting the length of the following cycle.

**Conclusion:**

Thus TRPM channels regulate the variability of the defecation oscillator in *C. elegans*. We conclude that the mean and the variability of the defecation oscillator are separable. Our results support the notion that there is a strong underlying pacemaker which is able to function independently of the observable defecation rhythm and is not perturbed by increases in the variability of the cycle.

The interaction of *gon-2 *and *gtl-1 *with other components of the oscillator shows that TRPM channels play an important role in the oscillator machinery. Such a role may be through either regulation of cation levels or membrane properties or both. Specifically our results support previous proposals that *gon-2 *and *gtl-1 *regulate IP_3 _signalling and that *kqt-3 *may act by altering calcium influx.

Our results provide novel insights into the properties of the defecation oscillator and thus to our understanding of ultradian rhythms.

## Background

**Figure 1:**
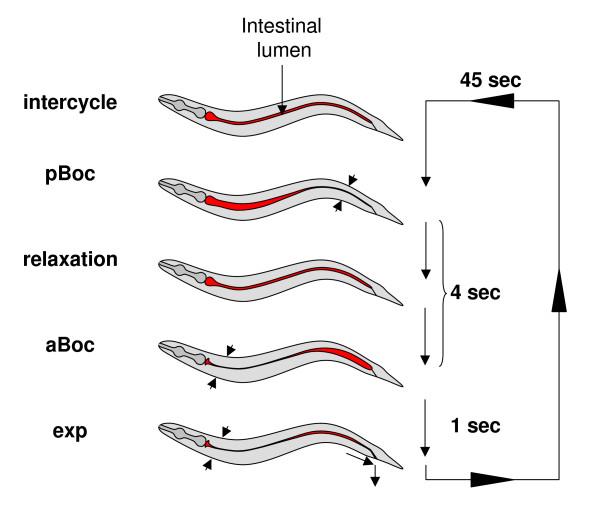
**The defecation cycle of *C. elegans***. A diagrammatic representation of the defecation cycle in *C. elegans*. Defecation depends on the successful execution of the defecation motor programme (DMP). Three readily observable steps in the DMP are: pBoc, posterior body wall muscle contraction, aBoc, anterior body wall muscle contraction and exp, expulsion. In normal undisturbed feeding animals this motor programme is initiated every 50 seconds with very high regularity. Underlying this rhythmic execution of the motor programme is an oscillator encoded in the intestinal cells of the animal. We use the term defecation cycle to refer to the cycle from one pBoc to the next. Figure after [40].

Ultradian rhythms, rhythms with a period of less than 24 hours, are a widespread and fundamental aspect of life. However, the genetic and cellular mechanisms that define the properties of many of these rhythms remain unclear. *Caenorhabditis elegans *has a number of ultradian rhythmic processes including defecation [1,2]. Defecation is a well defined and tractable model for the control of rhythmic behaviour [3,4]. In *C. elegans *defecation consists of a series of muscle contractions which make up the defecation motor program (DMP) (Figure 1). These can be divided into three readily visible steps: 1) contraction of the posterior body wall muscles (pBoc), which compresses the gut contents into the anterior part of the intestine; 2) contraction of the anterior body wall muscles (aBoc), which pushes the gut contents to the posterior part of the intestine; 3) contraction of the enteric muscles and relaxation of the enteric sphincter, which result in the expulsion of waste (Exp). This series of events takes place with a mean period (τ) of 45–50 seconds, in normal conditions at 20°C. The rhythm is tightly regulated with little deviation; in healthy wild-type worms the standard error of the mean (SEM) is typically 1 sec or less [1]. On the other hand, the DMP can be altered by environmental factors, such as temperature, food availability and mechanical stimulus [4]. Extensive genetic and other studies have identified a wide range of genes involved in this process including genes which alter the periodicity of the cycle (*dec *genes) [4,5]. In the prevailing model an oscillator underlies the DMP and causes the regular initiation of the motor program (Figure 1). For convenience we refer to this as the defecation oscillator. One important group of genes which influence the defecation oscillator are those involved in calcium signalling (see [4] for review). Of particular interest is the gene *itr-1 *which encodes the inositol 1,4,5-trisphosphate receptor (IP_3_R). IP_3_Rs release calcium from the ER in response to the production of IP_3_. *itr-1 *loss-of-function mutants have slower or absent defecation rhythms depending on their severity [6]. Mosaic and RNAi analysis strongly suggest that the function of *itr-1 *is in the intestinal cells [6,7]. The intestine of *C. elegans *consists of a single layered tube of 20 epithelial cells. Recent work has clearly demonstrated periodic calcium signals in posterior cells of the intestine and calcium waves that sweep in an anterior direction along the gut, thus further emphasising the importance of calcium in this process [6-10]. Calcium waves progressing in the opposite orientation have also been observed [8].

In this work we focus on the role TRPM (Transient Receptor Potential Melastatin) channels [11,12]. TRPM channels are a subgroup of the TRP channel superfamily [11,12]. Mammalian genomes encode eight members of the TRPM family, which can be divided into two groups consisting of TRPM1,3,6 and 7 and TRPM2,4,5 and 8. TRPM channels have a range of biophysical properties exhibiting both selective and non-selective cation permeation. TRPM6 and 7 have been implicated in magnesium homeostasis in the intestine and kidney [13-18] and mutations in TRPM6 underlie inherited defects in magnesium and calcium uptake [13,16]. *C. elegans *has three members of the TRPM channel family, *gtl-1*, *gtl-2 *and *gon-2*. *gon-2 *is known to be involved in the regulation of mitosis during gonadogenesis [19-23]. *gon-2*;*gtl-1 *double mutants have a severe growth defect which is ameliorated by the presence of high (typically 40 mM added Mg^2+^) in the growth medium [24]. Furthermore it has been shown that *gtl-1 *double mutants have reduced Mg^2+ ^levels in the intestine. Thus it has been suggested that these two genes have a key role in the regulation of the uptake of magnesium and other cations in the intestine [24].

Recent work has shed light on the function of *gon-2 *and *gtl-1*. Teramoto *et al*. suggested, based on genetic, functional and electrophysiological data, that *gon-2 *and *gtl-1 *function as separate channels with *gon-2 *contributing to a current with similarities to the previously characterised I_ORCa _[25]. More recent analysis suggests that *gon-2 *and *gtl-1 *are both required for I_ORCa _activity, that the channel is calcium selective and that channels encoded singly by *gon-2 *or *gtl-1 *have very similar properties. There are several other differences between the results from these two groups the causes of which remain unclear (see discussion in [26]).

Teramoto *et al*. [24] also showed that *gtl-1 *and *gon-2;gtl-1 *double mutants have a disrupted defecation cycle under certain conditions [24]. However successful culture of *gon-2;gtl-1 *double mutants requires that they are grown in unusually high Mg^2+ ^(typically 40 mM added Mg^2+^) compared to normal *C. elegans *culture conditions of 1 mM added Mg^2+^. As a result, experiments are performed either on worms in 40 mM Mg^2+ ^or on worms which have been shifted from 40 mM Mg^2+ ^to other conditions, thus exposing worms to either abnormally high levels of magnesium or large rapid changes in cation level. This may result in compounding various effects on the defecation cycle. Recently Xing *et al*., have also shown that *gon-2 *and *gtl-1 *mutants disrupt the defecation cycle [26].

In this work we show, using RNA mediated interference (RNAi), that in normal Mg^2+ ^conditions, ablation of *gon-2 *and *gtl-1 *substantially increases the variability of the defecation rhythm without significantly altering the period of the cycle. Therefore, we hypothesise that these genes function to control the variability (i.e. the rhythm) of the cycle. We show by analysing the behaviour of defective *gon-2(RNAi);gtl-1(RNAi) *worms at different temperatures and in *clk *mutants that this increase in variability is independent of changes in the period. Further we show that this increase in variability can be suppressed under certain genetic condition. Thus we propose that period and variability can be independently regulated. We also show that *gtl-1 *and *gon-2 *interact with the *itr-1*, *flr-1 *and *kqt-3 *genes, which also affect the variation and the rate of the defecation oscillator.

## Results

### The TRPM channels *gon-2 *and *gtl-1 *are required for a rhythmic defecation cycle

We set out to understand the nature of the input of TRPM channels into the defecation oscillator. To circumvent the problems associated with culturing worms at high magnesium (see introduction) and further define the role of TRPM channels in the defecation rhythm we used RNAi. Knock down by RNAi does not require that the worms are fertile (*gon-2 *null animals are sterile) and RNAi animals grow sufficiently well in normal culture conditions for analysis (CSMK unpublished).

**Figure 2:**
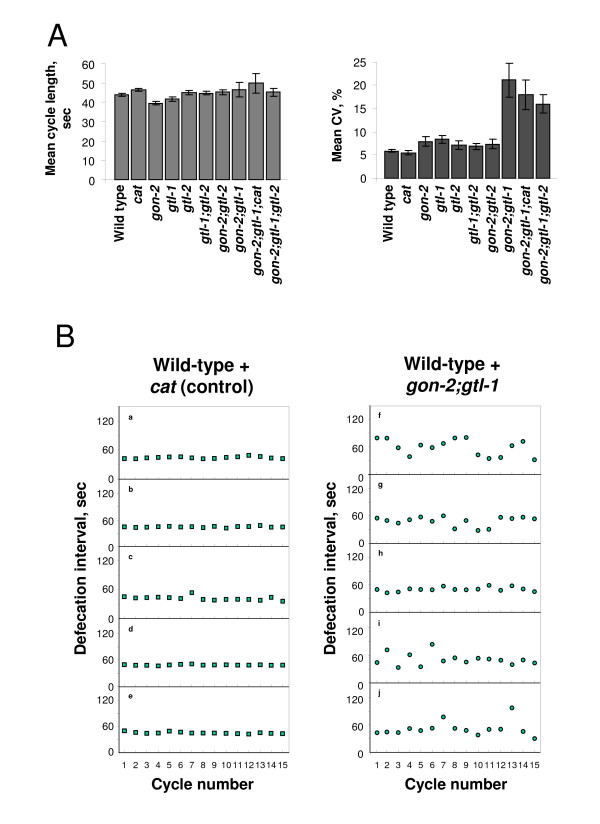
**Ablation of *gon-2 *and *gtl-1 *by RNAi increases the variability of the defecation cycle**. Members of the *C. elegans *TRPM channel gene family were knocked down using RNAi. When *gon-2 *and *gtl-1 *are ablated together the variability (C.V.) but not the period is altered. **A: **The mean period for each population of worms and the mean coefficient of variation (C.V.). Error bars are SEM. **B: **The period of 15 consecutive defecation cycles for representative controls (a-e) and *gon-2(RNAi);gtl-1(RNAi) *(f-j) worms.

To measure the behaviour of the defecation oscillator we measured the duration from one pBoc (posterior body contraction) to the next. We used a protocol designed to minimize variation resulting from life stage, environment and temperature to score the defecation cycle (F Kippert, Pers. Comm.). As a control we injected animals with dsRNA for the chloramphenicol acetyltransferase (*cat*) gene. Experiments were performed at 20°C unless otherwise noted. We tested the effect of RNAi on each of the three TRPM channel proteins individually and in combination (Figure 2A). Overall RNAi of TRPM channels genes caused little change in the mean period of the defecation cycle. However we noted that simultaneous RNAi of *gon-2 *and *gtl-1 *(henceforward referred to as *gon-2(RNAi);gtl-1(RNAi)*) resulted in a significant increase in the standard deviation. We therefore compared the mean of the coefficient of variation for the worms in each group (Figure 2A). It is clear that all combinations that include both *gtl-1 *and *gon-2 *show a substantial and significant increase in this parameter (P < 0.0001). We observed the same effect when we performed *gon-2 *RNAi on a *gtl-1 *knock-out strain HB201 *gtl-1(ok375) *[see Additional file 1]. The defecation oscillator is encoded in the intestinal cells of the animal [6,7]. In agreement with previously published data [24] we confirmed that *gtl-1 *is expressed in the intestine (by GFP fusion and *in situ *hybridisation) and that *gtl-2 *is not [see Additional file 1].

In *gon-2(RNAi);gtl-1(RNAi) *knock-down worms, the mean period of the cycle remains unaltered whilst the variability of the cycle is substantially increased (Figure 2A). Thus the mean and the mean C.V. for the control animals are 46.4 s and 5.5%, whereas those for *gon-2(RNAi);gtl-1(RNAi) *animals are 46.5 s and 21.1%. That is, the variability has increased by nearly four fold. Examples of the pattern of defecation periods in individual worms (figure 2B) show that individual worms have increased variability, excluding the possibility that this effect results from variable levels of RNAi. This strongly suggests that *gon-2 *and *gtl-1 *function together in regulating the rhythmicity of the cycle.

### The arrhythmicity conferred by *gon-2(RNAi);gtl-1(RNAi) *knock-down is independent of the mean of the rhythm

Our results suggest that the increase in variability caused by knocking down *gon-2;gtl-1 *function is largely independent of the mean. To test whether mean period and variability could be manipulated independently we perturbed the mean of the defecation oscillator using environmental and genetic treatments.

**Figure 3:**
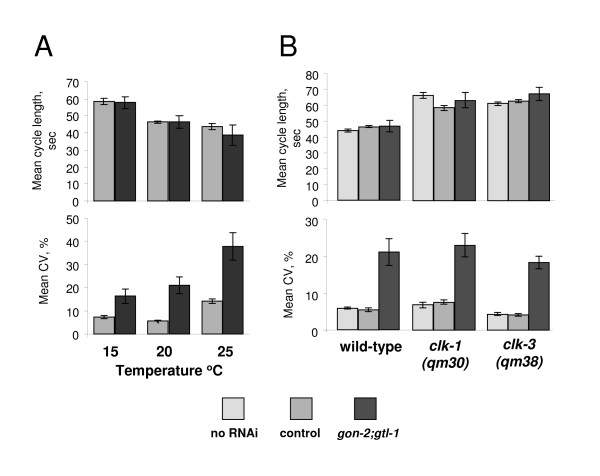
**The variability and mean of the defecation cycle can be altered independently**. The mean of the defecation cycle was perturbed using environmental and genetic approaches. **A**: The effect of temperature on wild type and *gon-2(RNAi);gtl-1(RNAi) *animals. The mean period and mean of the C.V. are shown. Worms were grown and assayed at the test temperature. **B**: The effect of genetic mutations, *clk-1 *and *clk-3*, which alter the mean. For each genotype we assayed untreated worms (labelled no RNAi), worms that had been injected with dsRNA for the *E. coli cat *gene (control) and worms injected with dsRNA for *gon-2 *and *gtl-1*. The mean cycle length and the mean of the C.V. are shown. Error bars are SEM in all panels.

First we tested the effect of temperature. We tested animals which were both grown and assayed at the test temperature (Figure 3A). At 15°C, 20°C and 25°C, *gon-2(RNAi);gtl-1(RNAi) *has no significant effect on the mean but in all cases results in a change to the mean C.V. of at least two fold (Figure 3A). These experiments suggest that the mean of the period is independent of the action of *gon-2(RNAi);gtl-1(RNAi)*. This can be seen by comparing the results at 15°C and 20°C. In these experiments the mean at 15°C is increased in both sets of animals to about 59 seconds. Thus *gon-2(RNAi);gtl-1(RNAi) *has no effect on the mean but alters the variability of the oscillator over a range of temperatures.

A wide range of mutants have been isolated which alter the period of the defecation oscillator (see [4] for review). *clk *mutants have changes in a range of rates and rhythms including life span and defecation. In *clk *mutants the defecation period is extended [27-29]. *clk-1 *encodes a putative hydroxylase required for ubiquinone biosynthesis [30] whilst the molecular nature of *clk-3 *is unknown. In our conditions, the period of defecation in both *clk-1 *and *clk-3 *mutants is increased by about 40%. We therefore knocked down *gon-2 *and *gtl-1 *by RNAi in *clk-1(qm30) *and *clk-3(qm38) *animals (Figure 3B). In both cases the period of defecation remained unaltered following RNAi, e.g. in the *clk-1 *animals it was 66 s whilst in *clk-1(qm30);gon-2(RNAi);gtl-1(RNAi) *animals it was 63 s. However in both cases RNAi of *gon-2 *and *gtl-1 *substantially increased the variability (see figure 3). In the case of *clk-1 *the C.V. was increased from 7 to 23% and in *clk-3 *from 4 to 18%. These experiments confirm that the period and rhythmicity of the process can be manipulated independently.

### TRPM channel knock-down interacts with other components of the defecation oscillator

We screened ten other genes which are known to be involved in the defecation programme for interactions with *gon-2(RNAi);gtl-1(RNAi)*. We detected strong interaction in four cases discussed below and no interaction with *crt-1(jh101), fat-3(wa22), kqt-2(ok732), lin-42(RNAi), unc-43(sa200) *and *unc-75(e950)*.

**Figure 4:**
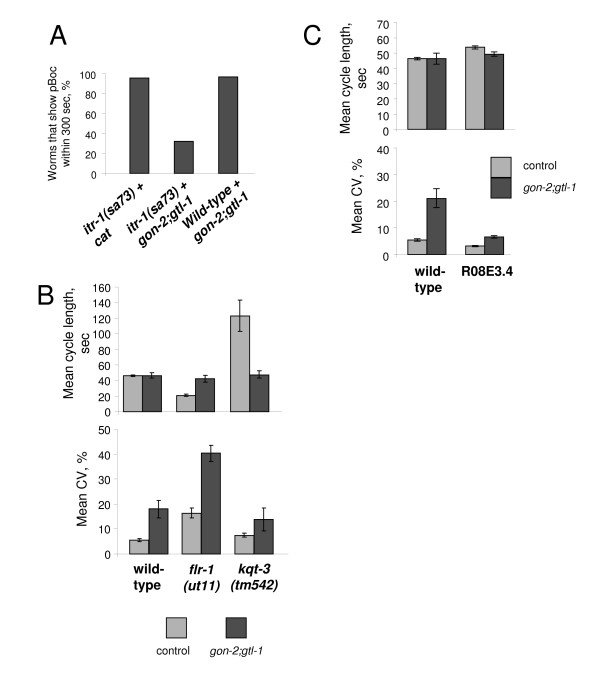
**Interactions of *gon-2;gtl-1 *with *itr-1, flr-1, kqt-3 *and *R08E3.4***. **A-B**: We tested for interaction of *gon-2(RNAi);gtl-1(RNAi) *with other genes known to be involved in control of the defecation cycle. **A**: When *gon-2 *and *gtl-1 *are knocked down in *itr-1(sa73) *mutant animals, the cycle is severely disrupted. The percentage of worms which successfully performed one or more pBocs within 300 sec is shown. **B**: *gon-2 *and *gtl-1 *were knocked down in a *flr-1(ut11) *and *kqt-3(tm542) *animals. For each genotype we assayed worms that had been injected with dsRNA for the *E. coli cat *gene (control) and worms injected with dsRNA for *gon-2 *and *gtl-1*. The mean cycle length and the mean of the C.V. are shown. **C**: The increase in variability in *gon-2(RNAi);gtl-1(RNAi) *animals can be suppressed by RNAi of R08E3.4. R08E3.4 was knocked down using RNAi, in combination with a control (*E. coli *cat) or *gon-2 *and *gtl-1*. The mean cycle length and the mean of the C.V. are shown. Error bars are SEM in all panels.

Loss-of-function mutants in *itr-1*, the gene that encodes the IP_3_R, disrupt the defecation cycle. The partial loss-of-function mutant, *itr-1(sa73)*, has an increased mean defecation period [5]. We tested whether the effect of *gon-2(RNAi);gtl-1(RNAi) *was altered in animals in which a component of the central oscillator is itself disrupted. In our conditions *itr-1(sa73) *animals show an increased mean (109 s) and also a very significantly increased variability (C.V. = 17%). This is similar to the data of Iwasaki *et al*. [5] and also to the effect of disrupting IP_3 _signalling using a dominant-negative approach [31]. We tested the effect of ablating *gon-2 *and *gtl-1 *by RNAi in these animals (Figure 4A). The result is severe disruption of the cycle, so that most worms fail to defecate at all. This is likely to be a synergistic effect: *gon-2;gtl-1 *knock-down does not affect the mean period thus an additive effect of *itr-1 *and *gon-2;gtl-1 *would produce a mean period close to that of *itr-1(sa73) *together with an increased C.V. In contrast we see an extreme disruption of the cycle.

*gon-2 *and *gtl-1 *encode putative plasma membrane cation channels [21,23]. We therefore tested whether they interact with other genes that are known to regulate defecation and to regulate plasma membrane ion flux. *flr-1 *encodes a member of DEG/ENaC sodium channel family [32]. *flr-1 *animals have a very fast defecation cycle [32] (mean 18 s in our conditions) (Figure 4B). They also have a very large variability (C.V = 19%). When we knocked down *gon-2 *and *gtl-1 *in these worms the variability showed a further very large increase (Figure 4B). In addition the mean cycle length was increased to close to the wild-type. Thus *gon-2(RNAi);gtl-1(RNAi) *suppresses the short defecation cycle phenotype of *flr-1 *animals and enhances the variability of *flr-1 *mutants. *kqt-3 *encodes a member of the KCNQ family of potassium channels [33] which is known to play a role in defecation [8]. *kqt-3 (lf) *animals have an increased mean and high variability. Reduction of *gon-2 *and *gtl-1 *in these animals causes a suppression of the increased mean with little or no effect on the variability (Figure 4B)

### RNAi of the zinc finger protein R08E3.4 suppresses the increased variability caused by reduction in *gon-2 *and *gtl-1 *function

We identified a further gene that modified the effect of reducing *gon-2 *and *gtl-1 *function Using RNAi we tested the effect of knocking down *lin-42 *(the *C. elegans *per homologue [34]) and a gene R08E3.4, which is also implicated in heterochronic regulation (Gardner and Rougvie pers. comm.). R08E3.4 is a zinc finger protein with similarities to the Ikaros family of transcription factors. *lin-42 *had little effect but, strikingly, R08E3.4 RNAi was able to substantially reduce the variability which results from *gon-2(RNAi);gtl-1(RNAi) *returning the variability to near wild-type levels (Figure 4C). The animals in these experiments showed the phenotypic effects (reduced fertility and growth) typical of *gon-2(RNAi)*;*gtl-1(RNAi) *ruling out the possibility that the reduction in variability was due to reduced levels of RNAi. Once again the mean of the period was unaffected. Thus the variability that results from *gon-2(RNAi)*;*gtl-1(RNAi) *can be suppressed by other genes.

### Analysis of *gon-2(RNAi);gtl-1(RNAi) *animals reveals self-regulation in the defecation oscillator

**Figure 5:**
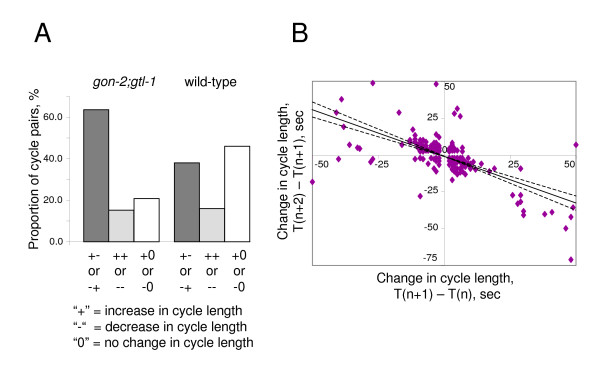
**Analysis of the defecation cycle in *gon-2(RNAi);gtl-1(RNAi) *animals reveals compensatory behaviour in the oscillator**. **A**: A histogram showing the frequency of the combinations of the direction of the change in cycle length for pairs of cycles. For each cycle we determined whether the period increased (represented by +), decreased (-) or remained constant (0). We then counted the number of cycle pairs which fell into each possible class. Those of classes "00", "0+" and "0-" are not shown. The frequency is shown as proportion of the total number of cycle pairs included in the analysis. **B**: A scatter plot of the change in period time in one cycle pair compared to the subsequent cycle pair for *gon-2(RNAi);gtl-1(RNAi) *animals. T(n) is the period of the cycle n. Linear regression was used to derive the line which, is shown with 95% confidence limits (dashed lines).

We observed that, in *gon-2(RNAi)*;*gtl-1(RNAi) *worms, increases in period length were often followed by decreases and vice versa (see for example Fig 2B panel i). We analysed the data from *gon-2*;*gtl-1 *animals to identify any such tendency. We classified the change from one cycle to the next (period cycle n+1 – period cycle n) as an increase if it was greater than 1 second (represented by "+"), a decrease if it was less than -1 secs ("-") or unchanged ("0") if it was between -1 and 1 sec inclusive. We then compared the number of pairs of cycles in which an increase was followed by a decrease or vice versa to the number in which an increase or decrease was followed by the same change (Figure 5A). We also quantified the number of pairs of cycles in which an increase or decrease was followed by no change. It is clear that there is a very strong tendency for increases to be followed by decreases and vice versa. Increases followed by decreases and the vice versa combination contribute equally to the numbers (not shown). Wild type animals show a similar bias but the close distribution to the mean makes it far less noticeable (Figure 5A). We next asked whether there was any correlation in the magnitude of a cycle and its following cycle. We compared the magnitude of the change on each cycle for all pairs of cycles in which, both cycles either increased or decreased by > 1 sec (Figure 5B). The result is a clear and significant (P < 0.0001) negative correlation (r = -0.6585 using a Pearson test). This suggests that larger increases or decreases tend to be followed by larger changes in the opposite direction.

## Discussion

The results presented in this paper demonstrate that disruption of the TRPM channels *gon-2 *and *gtl-1 *results in an increased variability in the defecation oscillator. By altering the mean, using mutations (in *clk-1 *and *clk-3*) and temperature, in *gon-2*;*gtl-1 *depleted animals we show that the variability of the oscillator and its mean can be manipulated independently. By testing interactions with other genes we identify informative interactions between *gon-2;gtl-1 *and *itr-1*, *flr-1 *and *kqt-3 *all genes known to be involved in defecation.

By using RNAi of *gon-2 *and *gtl-1 *we have been able to clearly define the major effect of knocking down these channels, in normal culture conditions. It is clear that reducing the activity of *gon-2 *and *gtl-1 *together results in a substantial increase in the variability of the rhythm without significantly affecting the mean. A similar pattern of disruption is seen in *inx-16 *mutants [9]. Importantly this increase in variability is independent of alterations in the mean caused by either genetic (*clk-1 *and *clk-3*) or environmental (temperature) manipulation. This suggests that the underlying oscillator is still oscillating in these mutants and that the mechanisms that set the period are also largely unaffected. It also suggests that *gon-2 *and *gtl-1 *are part of a mechanism that maintains regularity and therefore presumably the stability of the oscillator. It should be noted however that experiments using mutants of *gon-2 *and *gtl-1 *show that in these animals the period of the cycle is also altered [24,26]. This difference may reflect more severe impairment of channel function in mutant animals. We also found that in certain backgrounds, in particular those with disrupted membrane ion flux, *gon-2(RNA)i;gtl-1(RNAi) *also alters the mean period. We suggest that *gon-2 *and *gtl-1 *are part of a mechanism which is required for the stability of the oscillator in normal circumstances.

One possible explanation for the variability observed in the *gon-2(RNAi);gtl-1(RNAi) *animals stems from the suggestion that the period of the oscillator actually reflects an underlying faster pacemaker with a period of about 15 seconds, with suppression of intermediate calcium signalling events [8]. Thus in *gon-2;gtl-1 *animals it could be that they execute the DMP on random underlying pacemaker events. We did not detect such a trend; most of the variability appears to result from continuous spread about the mean. However we cannot rule out that some may reflect a changed response to the pacemaker as identifying this would require very large sample sizes.

How can we explain the observation that we can disrupt the rhythmicity of the cycle without disrupting the mean? One explanation for the ability of the animals to maintain a normal period but have increased variability is that there is a mechanism that sets the period and that the oscillator fluctuates around this input, so that disruption of *gon-2 *and *gtl-1 *compromises the ability of the oscillator to adhere to the input period. This is clearly the case to some extent as genes such as *clk-1 *change the mean of the cycle without destabilising the oscillator. Alternatively the oscillator may be self regulating and so able to maintain a normal period even when its stability is disrupted. Our analysis of the cycle suggests that this may also be the case. We observed that there is strong tendency for increases or decreases in the cycle length to be followed by inverse changes on the next cycle. We also observed that there is some correlation in the magnitude of such changes. This property could reflect a passive property of the system or could reflect an active mechanism to compensate for divergent cycles. Such a mechanism may exist to maintain the correct flow of material through the intestine. In normal worms the cycle is very tightly regulated, suggesting that the period of the cycle is important to the animal. That this is still the case in *gon-2(RNAi);gtl-1(RNAi) *animals again points to the machinery setting the period being largely intact.

In our experiments we detect a clear synergy between *gon-2*, *gtl-1 *and *itr-1*. *itr-1 *has been shown to be downstream of *plc-3 *in the defecation oscillator [7] and analysis of interactions between *gon-2 *and *gtl-1 *and *plc-3 *has strongly suggested a link between the TRPM channels and IP_3 _signalling [26]. Our results further support this model. One possible explanation for the synergy of *gon-2;gtl-1 *and *itr-1 *is that in *gon-2*;*gtl-1 *mutants calcium uptake is disrupted either directly or indirectly which results in depleted stores resulting in, either misregulation of *itr-1 *or altered levels of calcium release from the stores. Interestingly, Nehrke *et al *[8] observed that partial depletion of *sca-1*, the worm SERCA homologue, did not result in arrhythmia. This suggests that proper store filling is not a key determinant of rhythmicity. Thus this role for TRPM channels may be unlikely. Another possible explanation for the synergistic effect with ITR-1 is that Ca^2+ ^flux through GON-2 and GTL-1 maybe acting on ITR-1 directly to modulate its activity (see [26] for an extensive discussion of this idea) as IP_3_Rs are known to be regulated by calcium levels [35]. Finally Ca^2+ ^influx through GON-2 and GTL-1 may contribute directly to calcium waves induced by ITR-1.

The effect of depleting *flr-1 *or *kqt-3 *together with *gon-2 *and *gtl-1 *suggests that the functions of *gon-2 *and *gtl-1 *are linked to the properties of the plasma membrane. *flr-1 *mutants have altered means and rhythmicity. Simultaneous knock-down of *gon-2;gtl-1in flr-1 *mutants results in shifts in both the mean and the C.V. This suggests that either, *flr-1 *and *gon-2;gtl-1 *act in parallel to maintain rhythmicity or possibly that one acts on residual activity in the other to further disrupt its function. *flr-1 *encodes a putative plasma membrane DEG/ENaC type channel in the intestine and *gon-2;gtl-1 *are also likely to be plasma membrane cation channels. One possibility is that the interaction between these two channel types is mediated by alterations to the electrophysiological properties of the membrane. Other membrane components also alter defecation, for example *kqt-2 *and *kqt-3 *which are both members of the KCNQ M-type K^+ ^channels. We show that simultaneous depletion of *gon-2, gtl-1 *and *kqt-3 *results in suppression of the increased mean resulting from *kqt-3 *depletion. Thus *gon-2 *and *gtl-1 *maybe targets of *kqt-3 *action. Nehrke *et al*. [8] proposed that *kqt *channels exert their effect by regulating calcium influx. If *gon-2 *and *gtl-1 *act by contributing to I_ORCa _and thus calcium influx [26], then our results may support this model. We speculate that *flr-1 *also alters this process. However it is unclear why modulation of *gon-2 *and *gtl-1 *activity by these mechanisms would produce a change in the mean period when this does not occur on depletion in wild-type animals. It is clear that the interactions of these components require further analysis.

Finally we have identified a putative transcription factor, R08E3.4, depletion of which suppresses the *gon-2(RNAi);gtl-1(RNAi) *phenotype. The mechanism of this action is unclear. Further analysis of this interaction should provide new insights into the function of *gon-2 *and *gtl-1 *in defecation.

## Conclusion

The defecation cycle of *C. elegans *provides an example of an oscillatory mechanism that controls an ultradian rhythm. Using RNAi we show that two orthologs of the TRPM cation channel family are important component of the part of the mechanism which ensures regularity. We show that the oscillator is self adjusting with respect to period and that this ability is not disrupted in *gon-2 *and *gtl-1 *depleted animals. These results indicate that there is a strong underlying pacemaker which is able to function independently of the observable defecation rhythm and is not perturbed by disruption of the variability of the cycle.

We also show that *gon-2 *and *gtl-1 *interact with both calcium signalling molecules (*itr-1*) and other channels in the plasma membrane (*flr-1, kqt-3*). Thus the TRPM channels play an important role in the machinery of the oscillator. Our results are compatible with a model in which *gon-2 *and *gtl-1 *regulate IP_3 _signalling and in which *kqt-3 *(and possible *flr-1*) regulate *gon-2 *and *gtl-1 *function.

Our results provide a novel insight into the properties of the defecation oscillator and thus to our understanding of ultradian rhythms.

## Methods

### Worm husbandry and strains

*C. elegans *was maintained and cultured using standard conditions [36]. All worms were grown on NGM plates; containing 1 mM added MgCl_2 _and 1 mM added CaCl_2 _unless otherwise stated. We used the following strains: N2 (Bristol strain, wild type), MQ130: *clk-1(qm30*) [29], MQ131: *clk-3(qm38) *[37,38], JC55: *flr-1(ut11) *[38], JT73: *itr-1(sa73) *(a gift of J. Thomas) [5], TM542: *kqt-3(tm542) *(a gift of S. Mitani) and HB201: *gtl-1(ok375)*. HB201 is a derivative of VC244 out-crossed to N2 three times.

### RNA mediated interference

Double stranded RNA for RNAi was introduced by injection. Single stranded RNA for each strand was produced by *in vitro *transcription (MegaScript, Ambion) from clones containing approximately 1 kb of each cDNA amplified by PCR and cloned into pGEM-T (Promega). Details of clones are available on request. As a control dsRNA from the *cat *(chloramphenicol acetyltransferase) gene of *E. coli *was used. This was produced from the vector pPD136.60 (a gift of A. Fire). Preparation and injection of dsRNA was as described in [39]. Adult animals were injected, F1 progeny were then analyzed.

### Measurement and analysis of the defecation rhythm

Animals were grown, for at least two generations at the test temperature, normally 20°C. Animals were routinely cultured and measured on *E. coli *OP50. Analysis was performed in controlled temperature rooms. The length of the DMP was scored as the time between two posterior body wall contractions (pBoc) [See Figure 1]. To establish the parameters of the defecation rhythm we used the approach of Kippert (pers comm.). L4 worms were each scored for 15 consecutive cycles (unless otherwise noted) for each experiment. In most experiments more than 25 worms were scored and experiments were performed in duplicate or triplicate. Results from representative experiments are shown. Where appropriate the behaviour of untreated animals of a strain was confirmed as being similar to that previously reported.

For each data set we calculated the mean period of the defecation cycle and SEM. To measure the variation of the cycle we calculated the mean coefficient of variation which is the mean of the C.V.s for each worm in the experiment. The significance of differences in values was determined using unpaired t-tests.

### Statistical analysis of the defecation oscillator's behaviour

We defined the period of a cycle (n) as T(n). We defined an increase (represented by +) in cycle length as T(n+1)- T(n) > 1 sec. A decrease (represented by -) was defined as T(n+1)- T(n) < -1 sec. Values of ≥ -1 or ≤ 1 were defined as no change (0). In designing these criteria we assumed that a resolution of less than 1 sec in the difference was beyond the accuracy inherent in the experimental procedure. We defined nine possible combinations of the change in period between two cycles: 00, 0-, 0+, --, -0, -+, ++, +0, +-. To analyse a data set each pair of cycle differences was assigned to a particular class. We ignored members of the 00, 0+ and 0- classes as they are uninformative for this analysis. The numbers of members of the other classes were pooled as; "0-" & "0+", "++" & "--", "+-" & "-+". Results for a representative data set are shown. The original set contained 15 cycles for 25 worms yielding 325 cycle difference pairs.

To assess the correlation in magnitude between cycles we used the same data set. We determined the values of T(n+1)-T(n) for all pairs of period times. We censured all pairs in which either member of the pair had a magnitude of -1, 0 or 1. We then plotted T(n+1)- T(n) against T(n+2)-T(n+1). To assess correlation we used a Pearson test and we used linear regression (using GraphPad Prism) to derive the slope of the line. Significance was calculated using a two tailed T-test.

## Authors' contributions

CSMK, carried out the original defecation analysis on TRP channels and some interacting genes and helped to draft the manuscript. RPV–M carried out the analysis of interactions with further genes and played a substantial role in the preparation of the manuscript. SL assisted CSMK and RPV–M in the collection of data. KG refined the analysis of *gtl-1*. HAB conceived the study, participated in its design and coordination, performed data analysis and helped to draft the manuscript. All authors read and approved the final manuscript.

## Supplementary Material

Additional file 1 **Supplementary figures and legends, pdf, contains: **Supplementary figure 1: *gon-2(RNAi) *on *gtl-1(ok375) *worms increases variability in the defecation cycle. Supplementary figure 2: *gtl-1 *is expressed in the intestine whilst *gtl-2 *is expressed in the pharynx and excretory cell. Click here for file
