# Supplementary material for: TRPM channels are required for rhythmicity in the ultradian defecation rhythm of C. elegans
Source: BMC Physiol. 2008 May 21;8:11. doi: 10.1186/1472-6793-8-11 (PMC2409367; doi:10.1186/1472-6793-8-11)
Supplement: Additional file 1 — Supplementary figures and legends, pdf, contains: Supplementary figure 1: gon-2(RNAi) on gtl-1(ok375) worms increases variability in the defecation cycle. Supplementary figure 2: gtl-1 is expressed in the intestine whilst gtl-2 is expressed in the pharynx and excretory cell. [file 1472-6793-8-11-S1.pdf]

Supplementary Figure 1

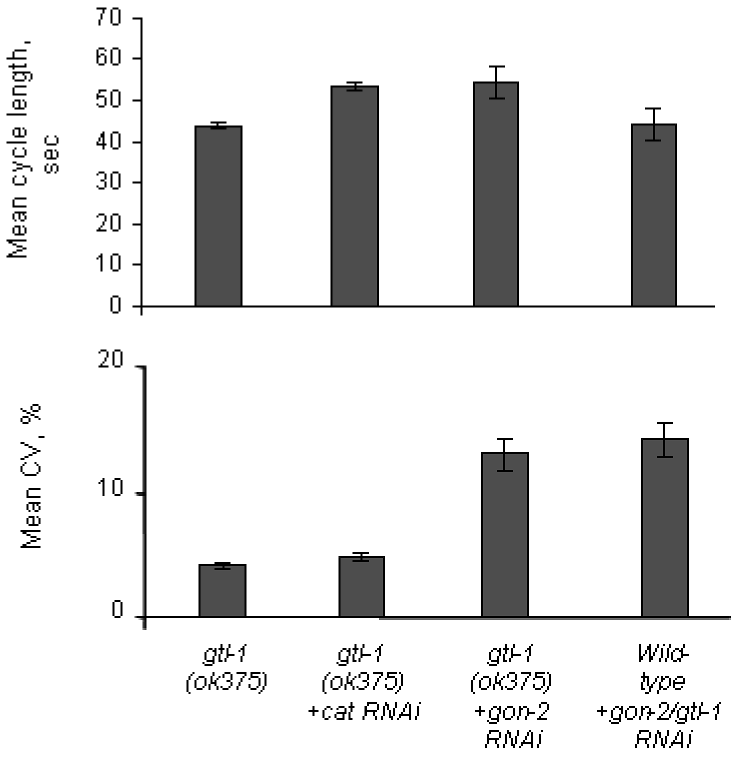

Supplementary figure 1. *gon-2(RNAi)* on *gtl-1(ok375)* worms increases variability in the defecation cycle. *gtl-1(ok375)* is a deletion derivative and putative null allele of *gtl-1*. The allele was outcrossed from the original strain (VC244) 3 times to produce HB202. RNAi was performed by the injection of dsRNA. dsRNA for the *E. coli* CAT gene was used as a control. The mean cycle length and the mean of the CV are shown. Error bars are SEM in both panels.

Supplementary Figure 2

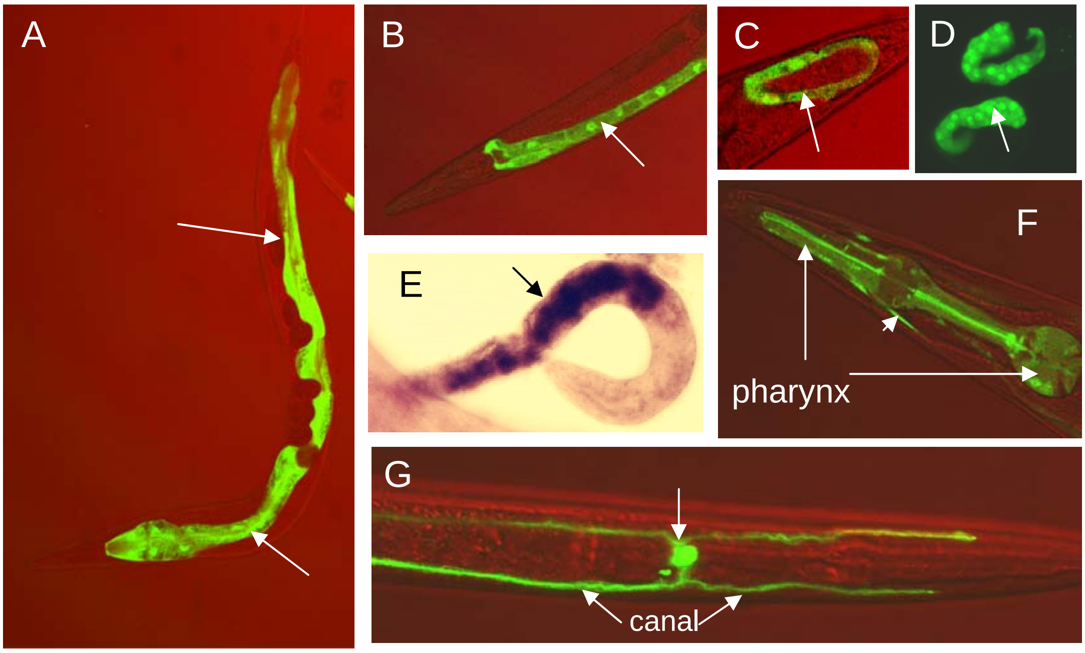

Supplementary figure 2. The *C. elegans* TRPM channels *gtl-1* and *gtl-2* are expressed in the intestine and pharynx and excretory cell respectively.

A-E: *gtl-1* is expressed in the intestine. The intestine is marked by white arrows in panels **A-D** and a black arrow in panel **E**. Panels **A-D** show transgenic animals carrying GFP fused to part of the *gtl-1* gene created as below. **A**, expression in the intestine (white arrows) of an adult. **B**, a closer view of the intestine (white arrow) of a young adult. **C** an oblique section in which GFP expression in the lining of the intestine (white arrow) is clear. **D**, expression in the intestines (white arrows) of two three-fold embryos. **E**, *In situ* hybridization of *gtl-1* in an L1 larva, **F,G**: *gtl-2* is expressed in the pharynx and excretory cell. A fusion of *gtl-2* and GFP was created in a similar way to *gtl-1*::GFP above (see below). **F**, expression in the pharynx GFP is observed throughout the muscle cells, parts of the excretory cell are also visible (arrowhead). **G**, expression in the excretory cell (white arrow) of an L2 larva. The canals are labeled. The animal shown in G did not show expression in the pharynx and was used for clarity.

GFP fusions for *gtl-1* were constructed as follows. The 5´ end of the *gtl-1* mRNA was determined by 5´ RACE (unpublished). A PCR fragment extending from 4.8 kb upstream of the ATG start codon to the start of exon 2 was subcloned upstream of

GFP in the vector pHAB200 [1] so that the *gtl-1* and GFP sequences were translationally fused. Similarly a PCR fragment extending from exon 30 to 2kb downstream of the stop codon of *gtl-1* was translationally fused downstream of GFP. The final plasmid, called pCK5 was microinjected into *C. elegans* [2] at 20 $\mu g \mu l^{-1}$ together with a *rol-6* marker [3] and *Pvu*II digested *C. elegans* genomic DNA (75 $\mu g \mu l^{-1}$). Stable transformants were selected and analyzed using a Leica SP1 Confocal Microscope. Three independent lines were analyzed. GFP fusions for *gtl-2* were created in a similar way. The upstream fragment extended from 5.4 kb upstream of the ATG to the middle of exon 3. The downstream fragment extended from exon 25 to 2.4 kb downstream. The final plasmid (pCK7) was injected into *C. elegans* at 20 $\mu g \mu l^{-1}$ with the *rol-6* marker. *In situ* hybridization was carried out using the method of Mitani *et al.*, [4] and Ogawa *et al*., [5].

References

1. Baylis HA, Furuichi T, Yoshikawa F, Mikoshiba K, Sattelle DB: **Inositol 1, 4, 5-trisphosphate receptors are strongly expressed in the nervous system, pharynx, intestine, gonad and excretory cell of *Caenorhabditis elegans* and are encoded by a single gene (*itr-1*).** *J Mol Biol*1999, **294:**467-476.

2. Mello C, Fire A: **DNA transformation.** *Met Cell Biol* 1998, **48:**451-482.

3. Kramer JM, French RP, Park E-C, Johnson JJ. **The *Caenorhabditis elegans rol-6* gene, which interacts with the *sqt-1* collagen gene to determine organismal morphology encodes a collagen.** *Mol Cell Biol* 1990, **10:**2081-2089.

4. Mitani S, Du H, Hall DH, Driscoll M, Chalfie M. **Combinatorial control of touch receptor neuron expression in *Caenorhabditis elegans*.** *Development* 1993, **119:**773-783.

5. Ogawa H, Harada S, Sassa Y, Yamamoto H, Hosono R. **Functional properties of the *unc-64* gene encoding a *Caenorhabditis elegans* syntaxin.** *J Biol Chem* 1998, **273:**2192-2198.
